# The Level and Factors Differentiating the Physical Fitness of Adolescents Passively and Actively Resting in South-Eastern Poland—A Pilot Study

**DOI:** 10.3390/children9091341

**Published:** 2022-09-01

**Authors:** Anna Puchalska-Sarna, Rafał Baran, Magdalena Kustra, Teresa Pop, Jarosław Herbert, Joanna Baran

**Affiliations:** 1 Institute of Health Sciences, Medical College, University of Rzeszów, 35-310 Rzeszów, Poland; 2 Regional Clinical Rehabilitation and Education Center for Children and Youth in Rzeszów, 35-301 Rzeszów, Poland; 3 Solution-Statistical Analysis, 35-120 Rzeszów, Poland; 4 Institute of Physical Culture Sciences, Medical College, University of Rzeszów, 35-326 Rzeszów, Poland; 5 Natural and Medical Center for Innovative Research, 35-310 Rzeszów, Poland

**Keywords:** active free time, adolescents, factors affecting physical fitness, passive free time, physical fitness, Zuchora test

## Abstract

Physical activity (PA) is defined as any bodily movement produced by skeletal muscles that requires energy expenditure. Due to civilization’s development, we can observe a global decline in physical activity which negatively affects the state of physical and mental health. The physical activity of children and adolescents is a counterpart to their physical fitness. There is also more frequent spending of free time in a passive way rather than actively. The aim of the study was to determine whether there are differences in the physical fitness of young people who rest passively in relation to those who rest actively. In addition, it was checked whether factors, such as age, weight, body height and BMI differentiate the level of fitness in adolescents. Study group: 25 boys and 25 girls declaring active leisure activities. Control group: 25 boys and 25 girls declaring passive leisure activities. Age of the respondents ranged from 11 to 15 years (Me = 13; SD = 1.23). The research used: the author’s questionnaire and the Index of Physical Fitness of K. Zuchora. The results were statistically developed. The youth who spend their free time actively were characterised by a higher level of physical fitness than their peers who choose passive recreation. The students with a higher BMI obtained worse results than the children with a lower body mass index. In both groups, slightly better results were obtained by girls. A significant relationship between age and results has been observed in the control group—the results increased with increasing age. The level of physical fitness is higher in active forms of recreation than in passive rest. Physical fitness tends to increase with age but decreases with increasing BMI. Girls are characterised by a higher level of physical fitness than boys.

## 1. Introduction

Physical fitness (PF) is defined as the current ability to perform motor activities that require the involvement of strength, speed, endurance, motor coordination (agility) and suppleness. It is not only a function of our locomotor system but also the basis for the proper biological functioning of the whole body. PF consists of motor features, such as muscle strength, speed of movement, endurance and flexibility of the body, which is sometimes also called suppleness or agility. These features have an impact and depend on our health. In order to assess the level of PF (not taking movement tests into account), the psychophysical properties and the features of the body structure are usually assessed [1,2].

The level of PF is also determined by sex and age, along with which human motor skills change. A baby starts to sit up in the fourth month of life, at the end of the first year—to walk. From that moment on, motor coordination develops intensively, reaching a relatively high level at the age of 4–5 (the so-called golden age of motor skills) [3]. Generally, boys at this age are more mobile than girls, but all children prefer active play rather than spending time, for example, watching TV [4]. This situation changes with age. Adolescents are capable of acquiring complex motor systems, and at the age of 12–14, they begin the stage of sexual maturation. This period often causes changes in PF, as well as regression of some motor skills. It is related to the action of hormones, modification of body proportions (mainly in the case of girls in whom hormonal fluctuations also contribute to a decrease in self-esteem, lack of acceptance of their own body, and thus—aversion to physical activity (PA)) and changes in BMI [3]. The research of the last decade shows that adolescents with a high degree of body fat and with a high BMI show a lower level of fitness, as well as worse results in endurance tests [5,6].

Special PF means the ability to perform movements that are mainly related to equipment specific to a given type of sport (apparatus, accessories). All types of fitness have an impact on the overall physical development of a person. Therefore, the largest pool of exercises is focused on general PF [7,8]. The level of PF is determined by simple tests that have been developed for specific populations, e.g., pupils, students, manual or white-collar workers, considering age categories and sex [9,10,11]. For people who practice competitive sports, separate tests with slightly higher criteria have been developed [12].

PF is closely related to PA. which is any body movement generated by skeletal muscles that requires energy consumption. Physical activity includes exercise and other activities that are performed in the form of play, housework, work and recreational activities. This activity has a large impact on shaping, maintaining and increasing fitness. In general, the more physically active we are, the more often and regularly we exercise, and the more physically fit we become. However, the effectiveness of the exercises performed is subject to the individual circumstances of each person’s body and is associated with genetic predisposition. The PA of children and adolescents is a counterpart of their physical fitness. It also has a significant impact on other spheres of life. From a biological point of view, appropriate physical activity shapes a healthy and physically fit individual, who reacts to environmental threats [13,14,15]. Exercise as a subcategory of PA is planned, structured, purposeful and repetitive [16].

There is scientific evidence that PA provides essential health benefits for children and adolescents [17,18,19]. This conclusion is based on observations in which greater PA was associated with an increase in beneficial health parameters. The documented benefits include increased physical fitness (both cardio-respiratory and muscle strength), increased bone density, reduced body fat and a reduced risk of metabolic and cardiovascular diseases. There is also a reduction in the occurrence of symptoms of depression. Maintaining regular PA that begins in childhood and continues into adulthood reduces the risk of death from cardiovascular and metabolic diseases in later years [20,21,22].

According to WHO recommendations, the PA of children and adolescents aged 5–17 should include play, games, sports, transport, recreation, physical education or planned exercises in the context of family, school and community activities [23] to improve cardiorespiratory and muscular fitness, bone health, cardiovascular and metabolic biomarkers, and to reduce symptoms of anxiety and depression:

1. Children and adolescents 5–17 years of age should spend at least 60 min a day in Moderate to Vigorous Physical Activity (MVPA).
2. Physical activity for more than 60 min a day will provide additional health benefits.
3. Most of the daily physical activity should be aerobic. Vigorous Physical Activity (VPA) activities, including those that strengthen muscles and bones should be practiced at least three times a week.

According to WHO, in 2010 81% of adolescents between 11 and 17 years of age worldwide were not sufficiently physically active. Adolescent girls were less active than their peers: 84% of girls did not meet WHO recommendations, and among boys—78% of adolescents [15].

Comparing the data obtained in two stages of the Polish research, a team from HBSC found statistically significant differences from 2014 to 2018 in the level of adolescents’ PA. Recommendations for MVPA are met by only 17.2% of adolescents, which is less than one-fifth of the studied population. There was also a clear negative trend in this period, i.e., a decrease in the percentage of adolescents meeting the WHO recommendations for moderate PA, from 24.2% in 2014 to 17.2% in 2018 [24].

Researchers emphasise that parents play an important role in creating appropriate habits of movement. However, only 44 percent of children engage in PA after school (e.g., cycling, walking) with their parents. On the other hand, 48 percent of children are driven to school by their parents by car [25].

Importantly, the levels of fitness appropriate to sex and age in children and adolescents tend to remain in adulthood, and their determination and strengthening are the basis for a physically active lifestyle throughout childhood, adolescence and adulthood [26,27]. Studies have shown that physically fit children were willing to engage in physical activity and maintain their behaviours during adolescence, while children who are less physically capable tended to be physically inactive during adolescence [28,29,30]. Children who are more physically fit have the fundamental skills required to participate successfully at different levels of PA. Therefore, they are more likely to keep PA within their field of interest [31].

The aim of the study was to determine whether there are differences in the physical fitness of young people who rest passively in relation to those who rest actively. In addition, it was checked whether factors, such as age, weight, body height and BMI differentiate the level of fitness in adolescents.

## 2. Materials and Methods

### 2.1. Participants

The study covered 100 pupils of the Municipal School Complex No. 3 in Krosno (Poland). After obtaining the written consent of the parents and children, the project began. The study group consisted of 25 girls and 25 boys. The control group also consisted of 25 girls and 25 boys. All children were examined in the spring period at the turn of April and May. All the subjects lived in the town.

The subjects were divided according to the way they spend their free time into two groups: people declaring active leisure (study group) and people declaring passive leisure (control group). By completing the questionnaire, the child answered the question about how they like to spend their free time: passively or actively. It was a subjective self-assessment of each child. Examples of passive activity (e.g., sitting in front of a TV set, in front of a computer) and active activity (e.g., cycling, walking, playing football) are given so that the child can easily notice the difference between the concept of passive and active leisure.

The criteria for inclusion in the study group are pupils aged 11–15 years, declared active way of spending free time, consent of the legal guardian to the child’s participation in the study, and informed consent of the pupil to participate in the study.

The criteria for exclusion from the research are disability or injuries in the lower limbs that make it impossible to take up PA, health contraindications for participation in the fitness test, the pupil feeling unwell during the test, withdrawal of the consent of the child or his/her guardian to participate in the study, even during the tests.

### 2.2. Research Tools

The study used the assessment of physical fitness with the use of K. Zuchora’s Physical Fitness Index, which consists of a six-step assessment of the pupil’s fitness [32]. This assessment checks:

1. Speed (sprint run on the spot with simultaneous clapping of hands under the knees—the result is determined by the number of claps within 10 s),
2. Jumping (long jump from the spot—distance measured in the pupil’s feet),
3. Shoulder strength (hanging on gymnastic wall bars: with both hands, one hand, both hands with a pull-up, for boys, additionally hanging with a pull-up and lowering, once with the right hand, once with the left hand, with holding for every 10 s—time measured in seconds),
4. Suppleness (bending the torso forward—the result is determined by the ability to reach as low as possible with the hands and touch the head to the knees),
5. General endurance (endurance run on the spot—time measured in minutes),
6. Strength of the abdominal muscles (performing transverse “scissors” while lying on the back—time measured in seconds/minutes).

Depending on their sex and the results of the individual attempts, pupils were assigned points (from 0 to 6) which were then added up and compared with the age-appropriate norms. In this way, the level of the children’s physical fitness was determined (Table 1 and Table 2).

### 2.3. Statistical Analysis

The Statistica 10.0 program (TIBCO Software Inc., Palo Alto, CA, USA) was used for statistical analysis. In the case of variables expressed on qualitative scales, the results were presented in the form of frequency distributions with percentage values, while for variables on quantitative scales, the basic measures of descriptive statistics were calculated: arithmetic mean, median and standard deviation. Compliance with normal distribution was tested using the Shapiro–Wilk test. Due to the fact that the variables did not meet the assumptions regarding the use of parametric methods (the distributions significantly deviated from the normal distribution), non-parametric methods were used to verify the hypotheses.

The Mann–Whitney U test was used for comparisons between the two groups, while Spearman’s rank correlation coefficient was used to analyse the relationships between the variables. Comparative analyses for the qualitative variables were performed using the Chi-square test. The significance level was assumed to be α = 0.05. The results were considered statistically significant when the calculated test probability p satisfied *p* < 0.05.

## 3. Results

The age of all subjects in the study ranged from 11 to 15 years of age. The average BMI of the subjects was 20.68. The shortest pupil was 1.40 m and the tallest was 1.80 m. The children’s body weight ranged from 33 to 90 kg (Table 3).

The analysis carried out using the Mann–Whitney U test showed statistically significant differences between the study group and the control group with regard to the final result of Zuchora’s test (*p* < 0.001). The comparison of arithmetic means and medians clearly shows that adolescents who actively spend their free time are characterised by a higher level of physical fitness than their peers choosing passive forms of recreation.

The analysis showed no statistically significant differences between girls and boys in the study group with regard to the obtained results, but the test probability p result is at a level tending towards significance. On the other hand, in the control group, there is a statistically significant difference (*p* = 0.003) between girls and boys in relation to the obtained results. On the basis of the arithmetic means and medians, it can be seen that in both groups girls obtained slightly better results (Table 4).

The comparison of the distribution of scores using the Chi-square test confirms the earlier observations that the study group is characterised by higher fitness (*p* < 0.001). In the group actively spending free time, the majority (58%) are adolescents with a good level of fitness, 28% are pupils with very good fitness, 10%—high, and only 4% obtained a satisfactory score (with no minimum scores). In the group preferring passive forms, as many as two out of three pupils (66%) are only sufficiently fit, 32% obtained the minimum score, and only 2% are good (with no very good or high scores) (Table 5).

The statistical analysis showed a very clear and statistically significant difference (*p* < 0.001) between the groups in the scope of the performed attempts. Pupils in the test group achieved significantly higher results in each of the individual attempts of Zuchora’s test than their peers in the control group (Table 6).

The analysis showed the existence of statistically significant differences (*p* < 0.001) between the study and control group in terms of body weight and BMI value. However, the differences between the groups with regard to age and body height were not statistically significant (*p* > 0.05). The analysis carried out with the use of the Mann–Whitney U test proved the existence of statistically significant differences (*p* = 0.042) between girls and boys in the study group in relation to the BMI value. However, the differences between sexes in relation to age, body height and body weight turned out to be statistically insignificant in this group (*p* > 0.05). In the control group, however, there were no statistically significant differences (*p* > 0.05) between girls and boys in terms of age, height and body weight. In the case of BMI, the value of the test probability p tends towards significance (*p* = 0.051) (Table 7).

The calculations made for all the subjects revealed statistically significant correlations between the final result of Zuchora’s test and body weight (R = −0.46; *p* < 0.001) and BMI (R = −0.49; *p* < 0.001). Children with a higher body weight or a higher BMI had lower results in Zuchora’s test.

Subgroup analysis allows for the discovery of differences in the relationships between the variables. In the group of active pupils, the final test result is correlated with the BMI value (R = −0.38; *p* = 0.006) as well as its components, body height (R = −0.30; *p* = 0.035) and body weight (R = −0 49; *p* < 0.001). As with the calculations for the entire sample, we are dealing with negative correlations of average strength. In the control group, only the relationship between age and results is significant (R = 0.34; *p* = 0.017), and its direction is positive, which means that older children had higher results in Zuchora’s test (Table 8).

## 4. Discussion

The aim of the study is to check to what extent the physical fitness of pupils spending leisure time actively differs from that of pupils spending their free time passively.

According to ECOG, it is necessary to explain the differences and similarities between physical activity and physical fitness. Of course, they are highly dependent and interrelated; however, they are two separate concepts that also need to be considered separately. Fitness depends on the level of physical ability (e.g., being able to participate in open-access physical education classes would require walking or running skills) [33].

Several studies have investigated physical activity and its effects on obesity and health, showing that regular physical activity combined with improved physical fitness reduces the risk of obesity and several metabolic problems (e.g., diabetes mellitus, metabolic syndrome, heart disease) and also improves overall health [34].

Vincent et al., Used the daily step counting method to assess physical fitness. The study covered three countries: Sweden, Australia and the United States. The authors found that students from Sweden had the highest level of physical fitness, bearing in mind the impact of different conditions in which students live. It also found that in Sweden, about 70 percent of students participated in extracurricular sports activities, and in the United States, only 20 percent of students were physically active in extracurricular activities [35].

The research shows that pupils who spend their free time actively are characterised by a higher level of physical fitness than their peers who spend their free time passively. There are statistically significant differences between the study group (active adolescents) and the control group (passive adolescents) in relation to the final result of K. Zuchora’s Physical Fitness Index. Most of the study group (58%) completed the test with a good result, 28% with a very good result, 10% with a high result, and only 4% of the group obtained a satisfactory result (with no minimum scores). In the control group (preferring passive forms of recreation) as many as two out of three pupils (66%) showed only sufficient fitness, minimum—as many as 32%, and only 2% were able to perform at a good level. In this group, no one received a very good or high score. Moreover, pupils in the test group obtained significantly better results in each individual attempt at Zuchora’s test than their peers in the control group.

These results are consistent with those of other authors. Children actively spending their free time dancing obtained much higher results in individual attempts of the test (the final results were very good and high, only two boys received a good score) than the group of boys who did not engage in any PA (the final scores were sufficient, with a smaller percentage minimum and good). The level of PF was assessed in both groups using K. Zuchora’s Physical Fitness Index [36].

Frömel et al., found that the most common forms of physical activity chosen by boys were team games, swimming and skateboarding, while in girls the most common forms of activity were swimming, dancing and skateboarding [37].

The authors’ own research also checked whether the test results depended on age, height, body weight and BMI. The calculations carried out for all the subjects showed statistically significant correlations between the final results of the physical fitness test and body weight and BMI. This means that body weight and BMI have an influence on fitness level. The analysis carried out separately in the study and control groups allowed for the observation of differences in the relationships between the variables. In the study group, the final result of the test was influenced by the BMI value, as well as its components, i.e., body height and body weight. Here too, the increasing values of the parameters determining body structure were accompanied by a decrease in the final values of Zuchora’s test. In the control group, there is only a significant relationship between age and results, and in this case, the test results increase with age. These results are consistent with the results of other researchers who found a statistically significant decrease in the level of children’s fitness in the study group with their height and BMI. Higher efficiency positively correlated with declarations of active forms of recreation. The authors assessed physical fitness with the use of Zuchora’s test [3].

Moreover, in another study 1/5 of pupils with excess body weight assessed themselves as not very active or physically inactive, 40% hardly or never attended physical education classes, and 1/3 did not systematically engage in any physical activity except PE classes. Comparing this group with peers with a normal BMI, obese junior high school pupils turned out to be significantly less physically active, and much less often participated in PE and sports outside school. However, no statistically significant differences were found between the groups in terms of the amount of time spent passively [38].

Based on the analysis of the results of Zuchora’s test, it turned out that there are no statistically significant differences between the girls and boys in the study group, but the test probability score is at a level tending towards significance. This means that the level of fitness of both sexes is at a similar level (although we can observe a slightly higher level in the girls surveyed). In the control group, however, there is a statistically significant difference between the sexes in relation to the results of Zuchora’s test—girls obtained better results than boys. Hoos et al., used other studies, claiming that there are no significant differences between the level of physical fitness of girls and boys; the level of energy spent on physical activity was the only difference between the sexes [39].

These results are consistent with the results of other researchers who, on the basis of PF tests carried out with children aged 11–15 years, found that the physical fitness of the pupils was at a satisfactory level. There were no clear differences in the obtained results between girls and boys, which indicated a similar development of the skills of all the pupils. The assessment of the pupils’ free time activity showed that they preferred spending free time actively [32].

As shown by Riddoch et al., European studies of children’s physical fitness show a higher level of fitness in boys than in girls [40].

Another test to assess your physical fitness is the Fitnessgram Battery Test. Studies carried out with its use show that men presented significantly higher results in the test of upper strength (*p* < 0.001) and aerobic capacity (*p* < 0.001), while women showed higher results in the sit and reach test (*p* < 0.001) in the torso lifting test (*p* < 0.005) and in the value of fat mass (*p* < 0.001) [41].

Standardised studies carried out using the EUROFIT test showed that Boys performed significantly (standardised differences > 0.2) better than girls in the tests of muscle strength, muscle power, muscle endurance, speed and CRF, but worse in the flexibility test. Physical function generally improved faster in boys than in girls, especially in adolescence [42].

In another study which used Zuchora’s PF Index more than half of the examined pupils undertook physical activity two to three times a week; 12% of the subjects declared low physical activity. Only 9% of the adolescents undertook physical activity five times a week or more. Pupils in this group showed better fitness in the test than those who practiced sports less frequently. Moreover, decreased physical fitness was found in the group of pupils with an excessive BMI index, and the examined girls showed higher fitness than boys. The analysis of the results proved that age does not significantly affect the level of physical fitness of the pupils [43].

Analysing our own research, it was found that in the study group there were statistically significant differences between girls and boys (*p* < 0.05) in relation to the BMI value. However, in relation to age, height and weight, the differences between the sexes in this group turned out to be statistically insignificant (*p* > 0.05).

In the control group, there were no statistically significant differences (*p* > 0.05) with respect to age, height, weight and BMI. However, in relation to BMI, the value of the test probability p tended towards significance (*p* = 0.051)—boys were characterised by a slightly higher body mass index. The better results obtained by girls may be a consequence of lower body mass indexes of girls as compared to boys.

In a large Greek cohort of 424,328 girls and boys aged 6–18 years, boys typically outperformed girls for cardiovascular endurance, muscular strength, muscular endurance, and speed/agility, but lower flexibility (all *p* < 0.001). Older boys and girls achieved better results than younger ones (*p* < 0.001). Physical fitness test scores peaked around age 15 for both sexes [44].

The boys’ fitness was better than that of the girls, with the exception of the sit down and press the back test, in which the girls performed better. Except for the sit down and back press test and the 10 × 5 m pendulum run test, physical fitness was significantly related to age. These results were obtained from a sample of 11,186 children and adolescents (5546 boys and 5640 girls) aged 10 to 15 years old and were assessed in the French national BOUGE study. Participants were tested for cardiorespiratory fitness, muscular endurance, speed, flexibility and agility with the following tests: the 20 m Pendulum Test, Rollback Test, 50 m Sprint Test, Sit and Bench Test, and the 10× Test 5-m shuttle test. Percentile values were estimated for French youth as a function of age stratified by sex using the Generalised Additive Location, Scale and Shape (GAMLSS) model [45].

As for results from our own research, the correlations between BMI and the results of the fitness test turned out to be very significant; the score decreased with an increase in BMI. Moreover, girls were shown to be better than boys in Zuchora’s test. This may be related to the average lower BMI of female pupils than male pupils. Free time physical activity significantly influenced the level of physical fitness in the children. Pupils who declared active recreation obtained significantly higher results in individual attempts of Zuchora’s test, compared to their peers whose way of spending their free time was almost completely passive.

Many authors also compare the level of physical fitness of students living in different regions. Loucaides et al., studied the physical fitness of students from rural and urban areas living in Cyprus, taking into account the summer and winter periods. The authors found that the children showed much greater physical activity in the summer, taking into account the place of residence of the children. Students from urban regions were slightly more active in winter than in rural areas, where they were more active in summer [46].

Children’s physical fitness is an extremely important aspect of their future health. The conducted research proved that physical fitness depends on whether we spend our free time passively or actively. That is why it is so important for parents to encourage their children to spend their free time actively while setting an example themselves.

It is worth emphasising that the literature does not contain many items of literature comparing the assessment of physical fitness using the Zuchora index. Many researchers describe PA under the concept of PF, which is a mistake because they are two different concepts.

### Limitations

The data are cross-sectional and do not allow for an analysis of cause-and-effect relationships. Consequently, we do not know the direction of the relationships found.

Another limitation is the small study group and the lack of a normal distribution of results, which prevents more advanced analyses.

The results may be limited to some extent because they were conducted in one school and the research sample was not an ideal representation of the child population.

## 5. Conclusions

The level of physical fitness is higher in the case of those undertaking active forms of recreation than in the case of those engaged in passive leisure. Physical fitness tends to increase with age but decreases with increasing BMI. Girls are characterised by a higher level of physical fitness than boys.

Future research should focus on more people and should be repeated annually. It would also be worth comparing the above results at different times of the year, or with a sports intervention for a certain period of time.

## Figures and Tables

**Table 1:** The level of the test execution and the score in points.

Sex	Minimum 1	Sufficient 2	Good 3	Very good 4	High 5	Excellent 6
**Test 1.**						
**F** **M**	12 claps 15 claps	16 claps 20 claps	20 claps 25 claps	25 claps 30 claps	30 claps 35 claps	35 claps 40 claps
**Test 2.**						
**F** **M**	5 feet 5 feet	6 feet 6 feet	7 feet 7 feet	8 feet 8 feet	9 feet 9 feet	10 feet 10 feet
**Test 3.**						
**F** **M**	Overhang AA 3 s overhang AA 10 s	Overhang AA 10 s Overhang A 10 s	Overhang A 3 s overhang and pull up 3 s	Overhang A 10 s overhang and pull up 10 s	overhang and pull up 3 s pull up A 10 s	overhang and pull up 10 s LA/RA
**Test 4.**						
**F** **M**	Ankle grip	fingers-toes	fingers-ground	fingers wide-ground/	hands-ground	head-knees
**Test 5.**						
**F** **M**	1 min 2 min	3 min 5 min	6 min 10 min	10 min 15 min	15 min 20 min	20 min 30 min
**Test 6.**						
**F** **M**	10 s 20 s	30 s 1 min	1 min 1.5 min	1.5 min 2 min	2 min 3 min	3 min 4 min

A—one arm; AA—double arms; F—female; M—male; LA—left arm; min—minutes; RA—right arm; s—seconds.

**Table 2:** Standards for individual age categories.

Marks	11–12 Years	13–15 Years
Minimum	6	6
Sufficient	11	12
Good	16	17
Very good	20	22
High	25	27
Excellent	29	31

**Table 3:** Characteristics of the studied children.

Variables	N	$\bar{x}$	Me	Min.	Max.	SD
Age [years]	100	13.02	13.00	11.00	15.00	1.23
Body height [m]	100	1.61	1.60	1.40	1.80	0.08
Body weight [kg]	100	53.79	50.00	33.00	90.00	12.52
BMI [kg/m^2^]	100	20.68	20.01	14.82	31.16	3.73

N—numbers of participants, Max.—maximum value, Me—median, Min—minimum value, SD—standard deviation, $\bar{x}$ —average value.

**Table 4:** Comparative analysis of the results obtained in the Zuchora test in both groups.

		Zuchora Test Results					
Group	Sex	N	$\bar{x}$	Me	SD	Z	*p*
Study	All	50	20.38	20	3.25	8.47	<0.001
Control	All	50	12.34	13	2.57		
Study	Female	25	21.16	21	3.22	1.79	0.073
	Male	25	19.60	19	3.15		
Control	Female	25	13.44	14	2.12	2.94	0.003
	Male	25	11.24	11	2.54		

N—numbers of participants, Max.—maximum value, Me—median, Min—minimum value, SD—standard deviation, $\bar{x}$ —average value, *p*—test probability, Z—U Mann–Whitney test value.

**Table 5:** Distribution of grades awarded on the basis of the Zuchora test result and the applicable standards.

	Zuchora Test Results					
Mark	Study		Control		All	
	N	%	N	%	N	%
Minimum	0	0.0%	16	32.0%	16	16.0%
Sufficient	2	4.0%	33	66.0%	35	35.0%
Good	29	58.0%	1	2.0%	30	30.0%
Very good	14	28.0%	0	0.0%	14	14.0%
High	5	10.0%	0	0.0%	5	5.0%
All	50	100.0%	50	100.0%	100	100.0%
Chi-sq. test	χ^2^ = 88.6; df = 4; *p* < 0.001					

N—numbers of participants, %—percent of participants, χ^2^—chi-squared test.

**Table 6:** Comparisons of the results obtained in individual attempts of the Zuchora test between the study and control groups.

	Zuchora Test Results						
Test	Study			Control			*p*
	$\bar{x}$	Me	SD	$\bar{x}$	Me	SD	
Test 1	3.66	4	0.85	2.58	3	0.84	<0.001
Test 2	3.90	4	0.89	2.84	3	1.00	<0.001
Test 3	3.20	3	1.28	1.62	1.5	0.78	<0.001
Test 4	3.78	3.5	1.39	2.22	2	1.37	<0.001
Test 5	4.10	4	1.34	2.18	2	0.90	<0.001
Test 6	1.74	2	0.94	0.90	1	0.65	<0.001

Me—median, SD—standard deviation, $\bar{x}$ —average value, *p*—test probability.

**Table 7:** Comparisons of age, weight, height and BMI between girls and boys in the study and control group.

Variables	Sex	N	$\bar{x}$	Me	SD	Z	*p*
**All**							
Age	Study	50	12.98	13.00	1.29	−0.36	0.717
	Control	50	13.06	13.00	1.19		
Body height	Study	50	1.60	1.60	0.08	−1.12	0.263
	Control	50	1.62	1.61	0.08		
Body weight	Study	50	48.04	47.00	7.67	−4.45	<0.001
	Control	50	59.54	59.50	13.79		
BMI	Study	50	18.79	18.36	2.05	−4.89	<0.001
	Control	50	22.56	21.93	4.09		
**Study group**							
Age	Female	25	13.00	13.00	1.22	0.12	0.907
	Male	25	12.96	13.00	1.37		
Body height	Female	25	1.60	1.60	0.08	0.42	0.677
	Male	25	1.60	1.58	0.09		
Body weight	Female	25	46.32	46.00	5.16	−1.48	0.138
	Male	25	49.76	48.00	9.34		
BMI	Female	25	18.19	18.07	1.46	−2.04	0.042
	Male	25	19.40	19.48	2.38		
**Control group**							
Age	Female	25	13.12	13.00	1.17	0.23	0.816
	Male	25	13.00	13.00	1.22		
Body height	Female	25	1.63	1.61	0.07	0.76	0.449
	Male	25	1.61	1.59	0.10		
Body weight	Female	25	56.96	54.00	12.75	−1.58	0.114
	Male	25	62.12	63.00	14.56		
BMI	Female	25	21.37	20.72	3.61	−1.95	0.051
	Male	25	23.74	23.88	4.26		

N—numbers of participants, Max.—maximum value, Me—median, Min—minimum value, SD—standard deviation, $\bar{x}$ —average value, *p*—test probability, Z—U Manna-Whitney test value.

**Table 8:** Correlations of the results in the Zuchora test with the parameters of children.

Group	A Pair of Variables	N	R Spearman	T (N − 2)	*p*
**All**					
	Zuchora & Age	100	0.01	0.12	0.901
	Zuchora & Body height	100	−0.13	−1.31	0.192
	Zuchora & Body weight	100	−0.46	−5.17	<0.001
	Zuchora & BMI	100	−0.49	−5.58	<0.001
**Study**					
	Zuchora & Age	50	−0.15	−1.06	0.293
	Zuchora & Body height	50	−0.30	−2.17	0.035
	Zuchora & Body weight	50	−0.49	−3.86	<0.001
	Zuchora & BMI	50	−0.38	−2.86	0.006
**Control**					
	Zuchora & Age	50	0.34	2.47	0.017
	Zuchora & Body height	50	0.15	1.03	0.310
	Zuchora & Body weight	50	0.06	0.39	0.699
	Zuchora & BMI	50	0.02	0.11	0.916

## Data Availability

Data available from the authors of this publication.
